# Supplementary material for: Comparative Genomics and Environmental Distribution of Large dsDNA Viruses in the Family Asfarviridae
Source: Front Microbiol. 2021 Mar 15;12:657471. doi: 10.3389/fmicb.2021.657471 (PMC8005611; doi:10.3389/fmicb.2021.657471)
Supplement: Supplementary file 1 [file Table_1.DOCX]

Supplementary Material

# Supplementary Data

**Supplementary Data 1**. Hits to the MAG as determined by blastp.

**Supplementary Data 2**. Protein annotation of the viral MAGs.

# Supplementary Figures


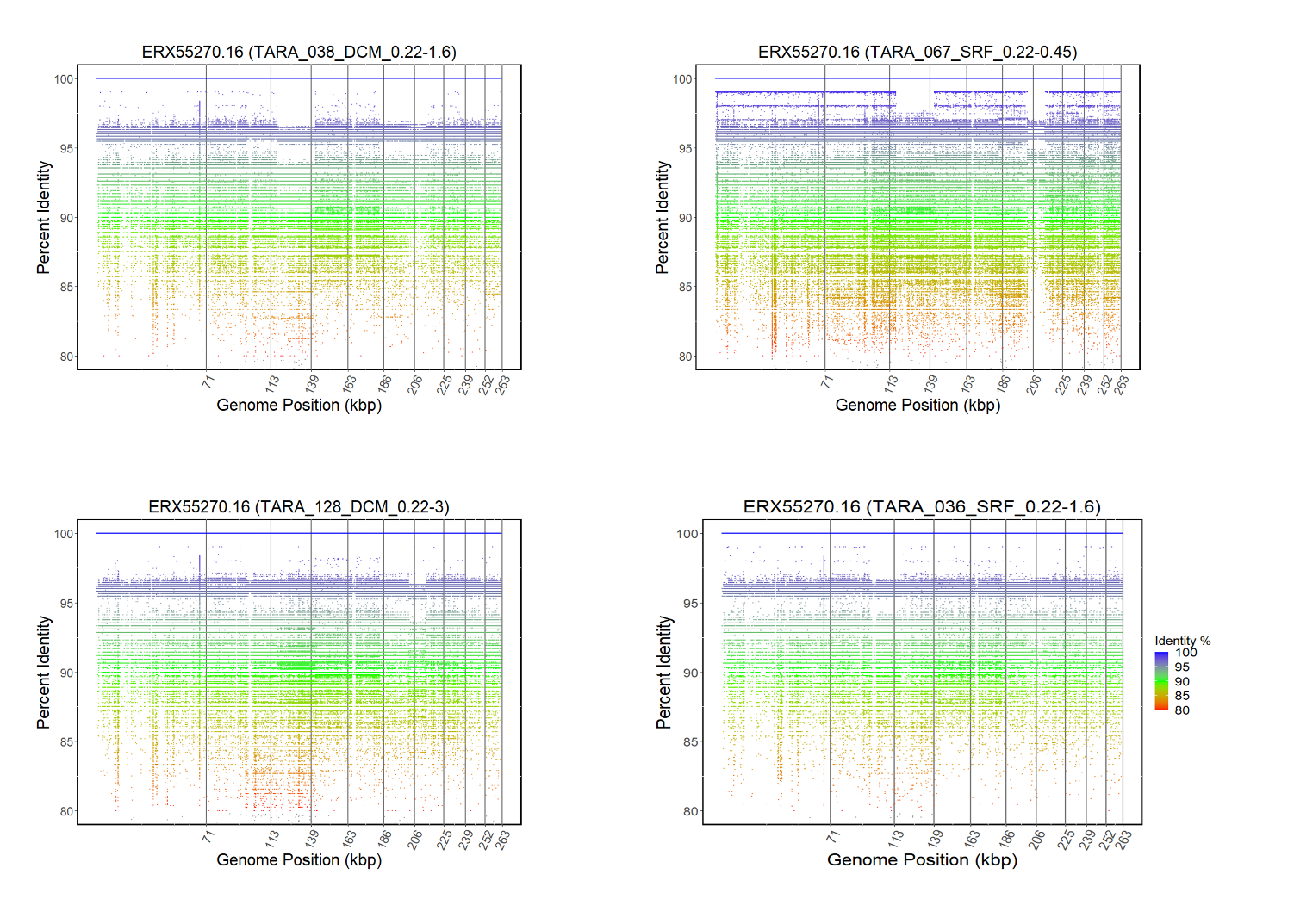


**Supplementary Figure 1.** Fragment recruitment plot for metagenomic reads to ERX552270.16. The x-axis of the recruitment plot shows position of the metagenomic reads along the genome length and y-axis represents the percent identity.


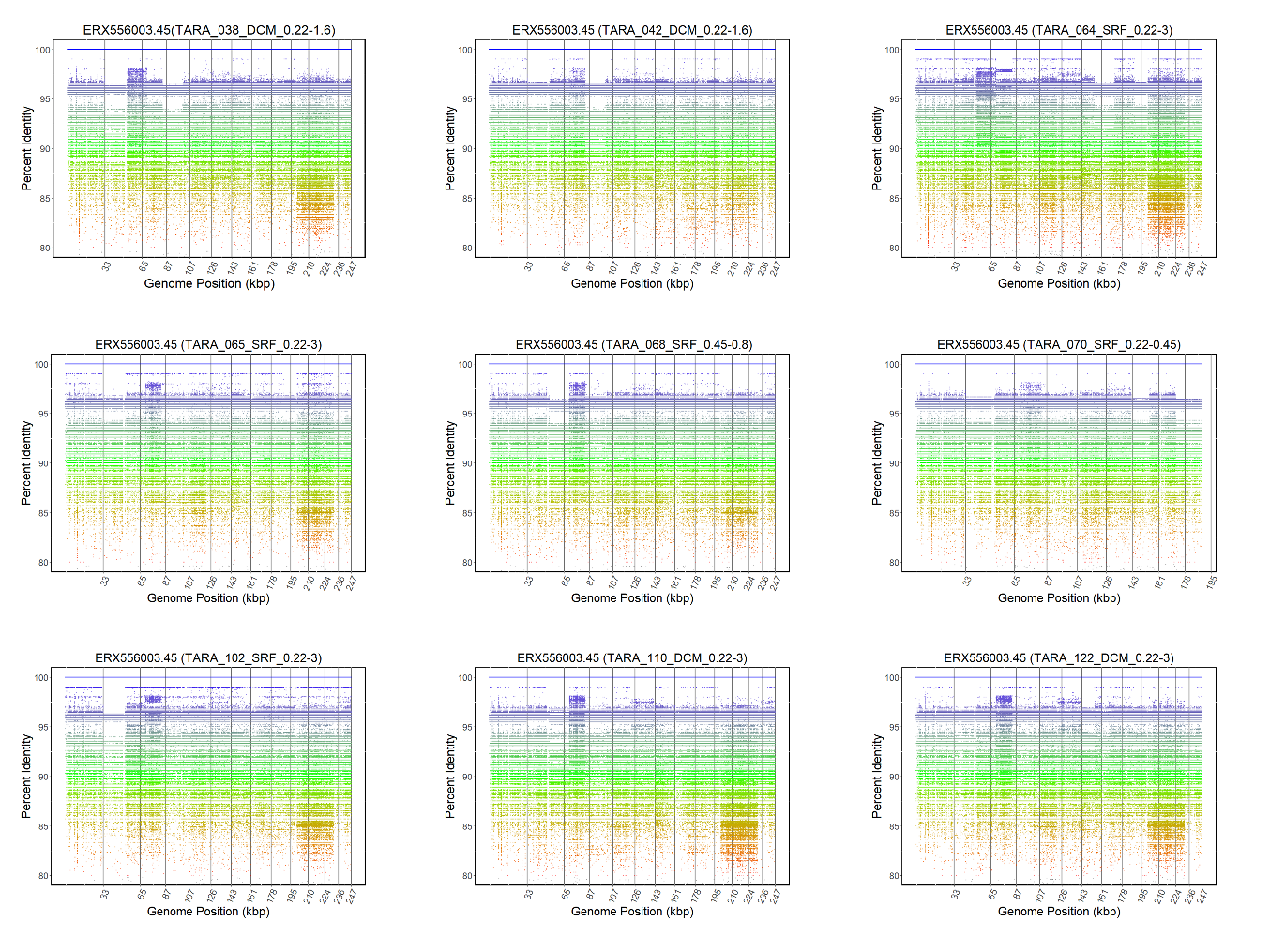

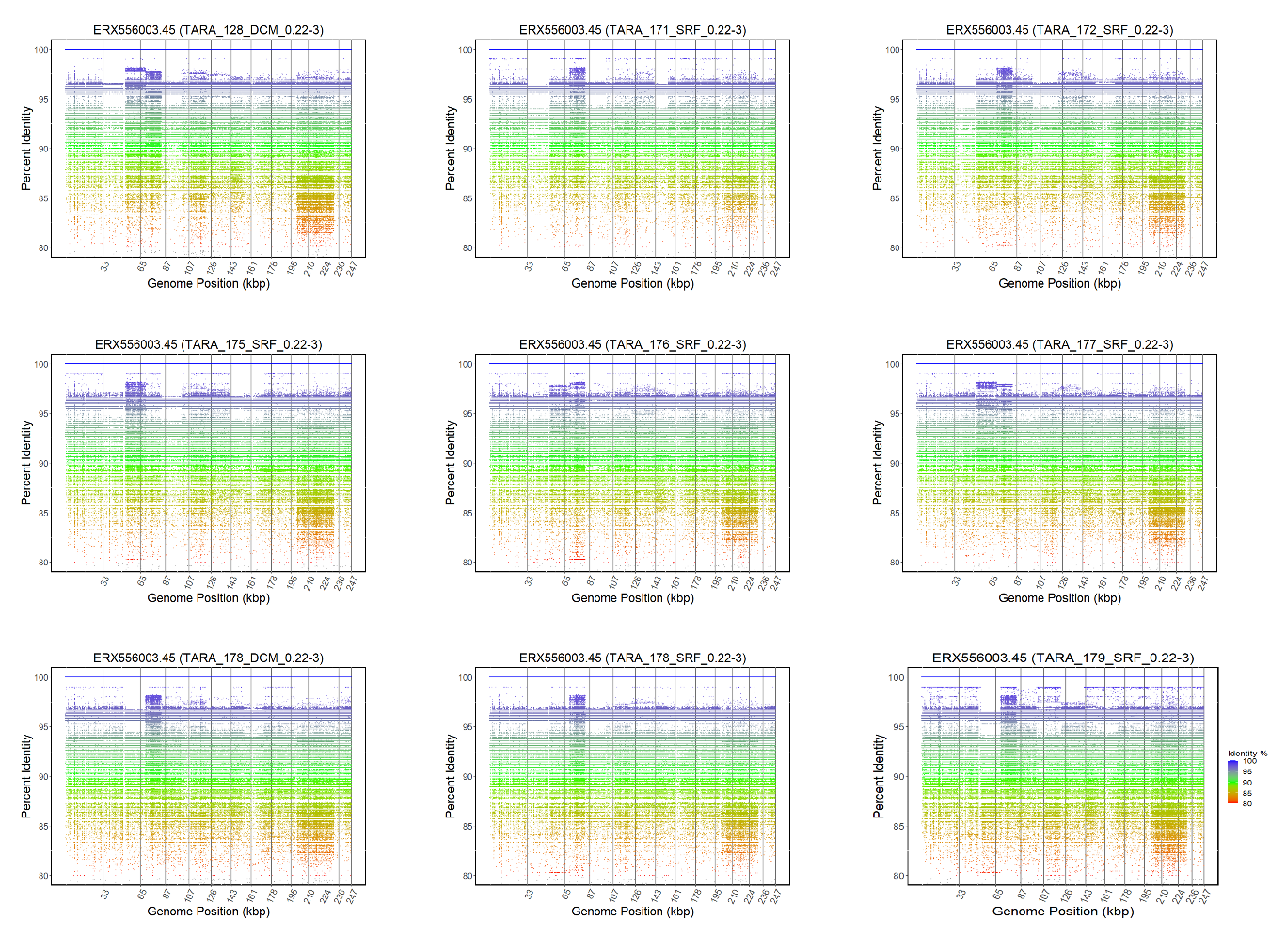


**Supplementary Figure 2.** Fragment recruitment plot for metagenomic reads to ERX556003.45. The x-axis of the recruitment plot shows position of the metagenomic reads along the genome length and y-axis represents the percent identity.
